# The 3-D structure of the Somma-Vesuvius volcanic complex (Italy) inferred from new and historic gravimetric data

**DOI:** 10.1038/s41598-017-07496-y

**Published:** 2017-08-16

**Authors:** Niklas Linde, Tullio Ricci, Ludovic Baron, Alexis Shakas, Giovanna Berrino

**Affiliations:** 1 Applied and Environmental Geophysics Group, Institute of Earth Sciences, University of Lausanne, Géopolis, 1015 Lausanne, Switzerland; 2 Istituto Nazionale di Geofisica e Vulcanologia, Via di Vigna Murata 605, 00143 Rome, Italy; 3 Istituto Nazionale di Geofisica e Vulcanologia, Osservatorio Vesuviano, Via Diocleziano 328, 80124 Naples, Italy

## Abstract

Existing 3-D density models of the Somma-Vesuvius volcanic complex (SVVC), Italy, largely disagree. Despite the scientific and socioeconomic importance of Vesuvius, there is no reliable 3-D density model of the SVVC. A considerable uncertainty prevails concerning the presence (or absence) of a dense body underlying the Vesuvius crater (1944 eruption) that is implied from extensive seismic investigations. We have acquired relative gravity measurements at 297 stations, including measurements in difficult-to-access areas (e.g., the first-ever measurements in the crater). In agreement with seismic investigations, the simultaneous inversion of these and historic data resolves a high-density body that extends from the surface of the Vesuvius crater down to depths that exceed 2 km. A 1.5-km radius horseshoe-shaped dense feature (open in the southwestern sector) enforces the existing model of groundwater circulation within the SVVC. Based on its volcano-tectonic evolution, we interpret volcanic structures that have never been imaged before.

## Introduction

**Figure 1:**
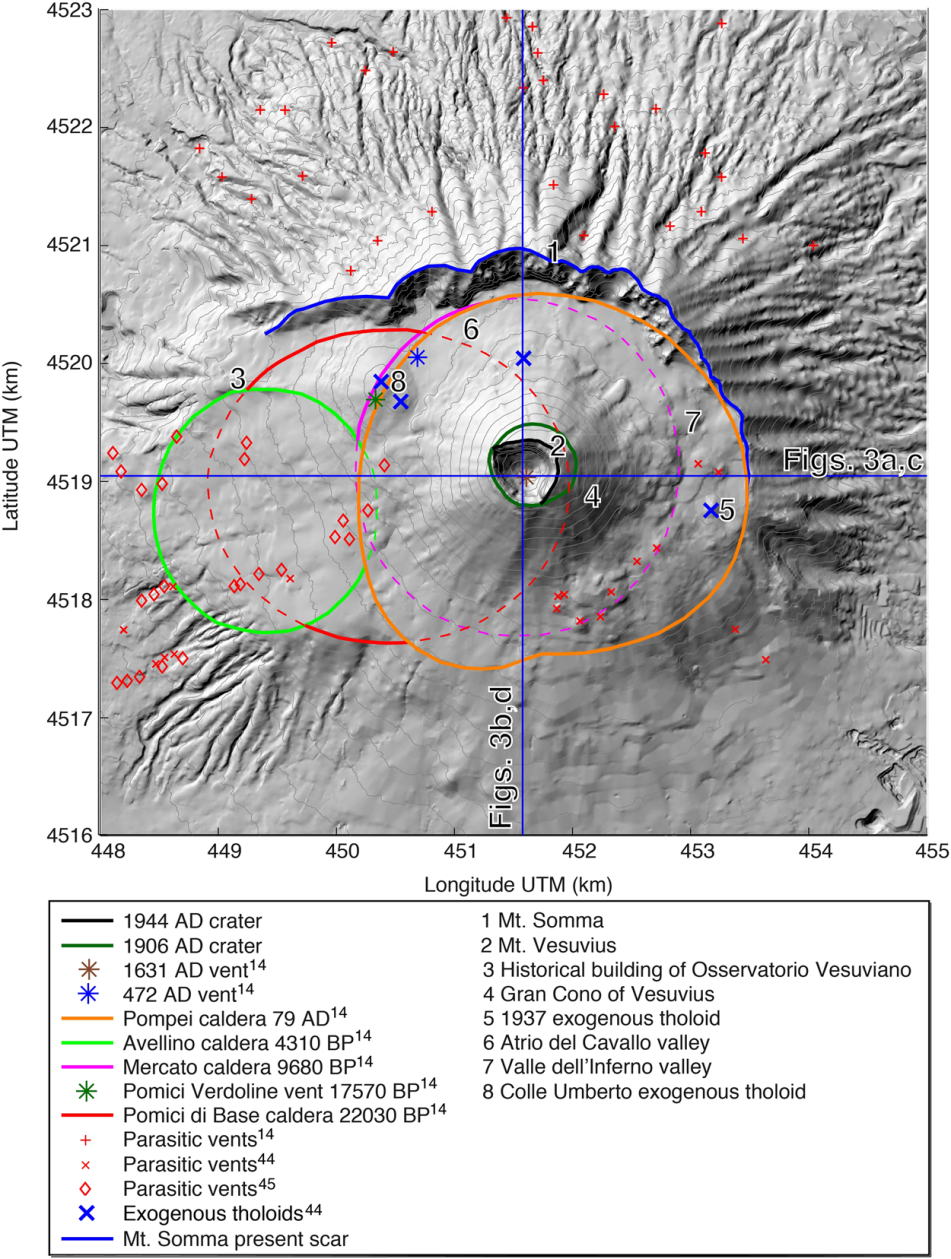
The main volcanic features of the SVVC^14, 44, 45^ with geographical names and topography produced from a digital elevation model^57^ using Surfer (version 14; http://www.goldensoftware.com/). Dashed lines refer to sectors of inferred plinian calderas that would have been removed by subsequent caldera forming events. The trajectories of the vertical sections shown in Fig. 3 are indicated by blue lines.

The Somma-Vesuvius volcanic complex (SVVC) is one of the volcanoes with the highest volcanic risk worldwide (it threatens 800,000 residents living on its slopes)^1–4^. The main volcanic hazards are pyroclastic flows and fallout, earthquakes, lahars, lava flows and floods. According to recent studies, the total economic impact of a subplinian eruption in the Vesuvian area, representing the reference scenario for the emergency plan^5, 6^, is estimated to 90 billion Euro^7^. The SVVC is a Quaternary composite stratovolcano located 15 km southeast of Naples (southern Italy) in the Piana Campana semi-graben structure. It is bordered by Mesozoic carbonate shelves at the intersection of northwest-southeast and northeast-southwest trending oblique-slip faults and east-west trending normal fault systems^8–11^. The history of the SVVC began 0.3–0.5 million years ago^12^ and is characterized by periods of closed conduit rest lasting up to 1000 years that are interrupted by plinian and subplinian explosive eruptions^13^. These eruptions display the same eruptive and syn-eruptive phenomena, but they differ in terms of the volume of emitted magma and the energy of the eruption^13^. The SVVC is composed of a multistage and older summit caldera (Mt. Somma, 1132 m a.s.l.) and a nested younger cone (Mt. Vesuvius, 1281 m a.s.l.) (Fig. 1). In the last 22 ka, four plinian caldera-forming eruptions (22 ka Pomici di Base, 9.7 ka Mercato, 4.3 ka Avellino, and AD 79 Pompei) and at least three major subplinian eruptions occurred (17.6 ka Pomici Verdoline, AD 472 Pollena, and AD 1631)^14^. Each plinian eruption produced a summit collapse that modified the dimensions and shape of the Mt. Somma caldera, presently characterized by steep walls in the northern sector and a gentle morphology in the southern one^15^. SVVC products include lava flows and pyroclastics emitted from summit craters and calderas, as well as from parasitic lateral vents, eruptive fissures and exogenous tholoids that are related to both explosive and effusive eruptions. Pyroclastic deposits of plinian and subplinian eruptions blanket the northeastern sector of Mt. Somma with thicknesses up to 70 m in topographic depressions on the lower slopes, while the southwestern sector of the SVVC is mainly covered by historic lava flows^16^. Since the AD 1631 subplinian eruption, preceded by about 500 years of rest^17^, Vesuvius entered in an open conduit phase that lasted until March 1944 when, after a violent Strombolian eruption, the volcano entered in a new close conduit quiescence.

Geophysical 3-D imaging may offer important insights about the history of the SVVC and provide geometric constraints on past magma feeding systems. Several 3-D gravimetric inversion studies have been performed in the surroundings of the SVVC^18–22^, but they strongly disagree among each other and some of them are inconsistent with respect to other geophysical findings. For example, it is expected that the high-velocity zone underlying the Vesuvius crater that is prominent in seismic investigations^23–25^ should correspond to a high-density body with a clear and unambiguous gravimetric signature. A central anomalous body is also evidenced by aeromagnetic data and can be explained by a central highly magnetized body that extends from the surface down to at least 2 km below the sea level^26^. Nevertheless, the highest resolution 3-D gravimetric inversion study to date^20^ largely dismisses the seismic findings based on a density model that features a low-density body below the Vesuvius crater. This result contrasts with another 3-D density model^21^ that indicates a central high-density body, but the sparse station distribution used in the latter study yields a poor model resolution. It is important to better constrain and understand the origin of this central anomalous body as most of the present-day seismicity is confounded along its boundaries^27, 28^. Magnetic studies^26^ suggest that its top is located at shallow depths below the crater, while seismic studies disagree about its upper limit (e.g., 1.5 km depth^29^ or in the near subsurface^18^). It is recognized that the top interface is poorly defined by available seismic data and that the absence of a high-velocity body at shallow depths might simply be an artifact of the initial model used in the inversion^29^. The actual geometry of this high-velocity body, its origin and constitution is still uncertain. It is well-established that it takes its origin at depths that are considerably larger than the depth to the underlying carbonate basement^23^, which strongly suggests that it has an intrusive magmatic origin. A survey of seismic and seismological studies at other volcanoes worldwide reveals that central high-velocity bodies are commonly interpreted as magmatic intrusions^23^. Initially, it was suggested that the high-velocity body was made up of slowly cooled magmatic dikes^30^, while later work has argued that such an interpretation is incompatible with the time-scales associated with the magma cooling process^25^. Instead, an alternative explanation was proposed in terms of very fast solidification by magma quenching^25^.

Using new (and old) gravimetric data and a recent inversion algorithm conceived for volcanic targets^31^, we present the most detailed density model of the SVVC to date. We have acquired and processed gravimetric data from 297 new stations (precisely positioned using differential Global Positioning System (DGPS)) with locations chosen to complement previous studies. Furthermore, we cover the summit area in much more detail than previously (the data set include the first gravimetric data within the Vesuvius crater). In the inversion, we account for the topography by incorporating a modern digital elevation model (DEM) based on light detection and ranging (LIDAR) data at a resolution of 5 m. We present this detailed 3-D density from the surface down to depths of 1–2 km. The main objectives of this study are to (1) provide a definite answer concerning the presence or not of a dense body underlying the Vesuvius crater and (2) interpret our high-resolution 3-D density model in terms of the evolution and present structure of the SVVC.

## Results

**Figure 2:**
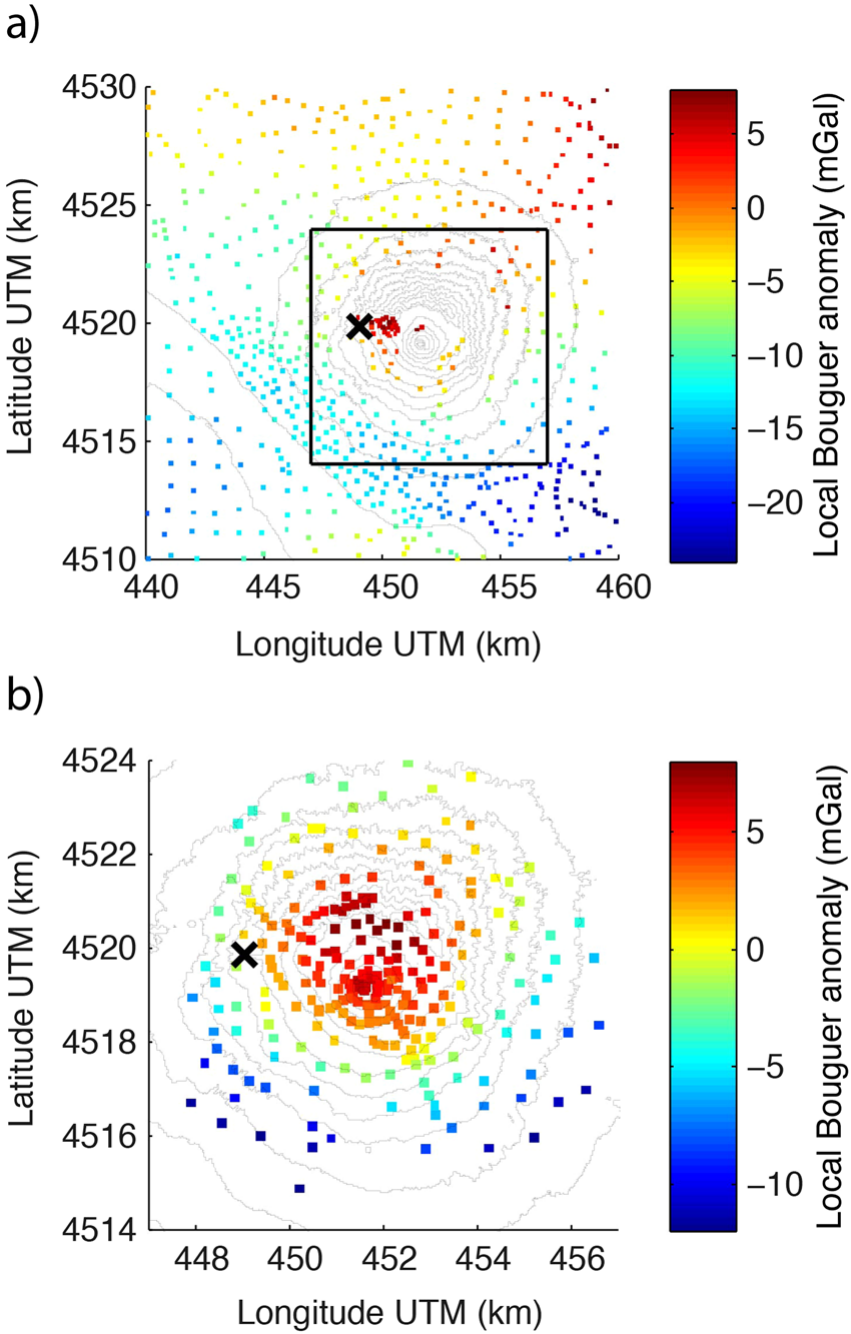
Local Bouguer anomaly of the (**a**) historic data and (**b**) new data set constructed using a density of 2200 kg m^−3^. The region in (**b**) is represented by a black square in (**a**). The values are given with respect to the reference station of the new data set (indicated with a ×-sign). The maps were created using Matlab (version R2013b; www.mathworks.com).

Local Bouguer anomalies provide a first visualization of the available gravimetric data (Fig. 2). In agreement with previous work^32^, we used a density of 2200 kg m^−3^ for topographic and Bouguer plate corrections. We referenced the local Bouguer anomalies to a base station that was located close to the historical building of the Osservatorio Vesuviano (Fig. 1). From these data, we removed a regional trend that was inferred as part of the inversion process. Figure 2a presents the distribution of the historic data in a square of size 20 km × 20 km that is centered on the Vesuvius crater. These data (and additional data outside this region) have been used in previous 3-D inversions^21^. Our new data set was acquired within the central 10 km × 10 km square region shown in Fig. 2a. It is clear that the coverage of the historic data is poor in this central region, partly because of the difficult access. The coverage of our new data set (Fig. 2b) is comparatively homogeneous with a refinement in the summit area. This new data set also includes the first-ever gravity measurements in the Vesuvius crater. The new local Bouguer anomalies (Fig. 2b) clearly highlight that the central area of the SVVC is denser than 2200 kg m^−3^.

**Figure 3:**
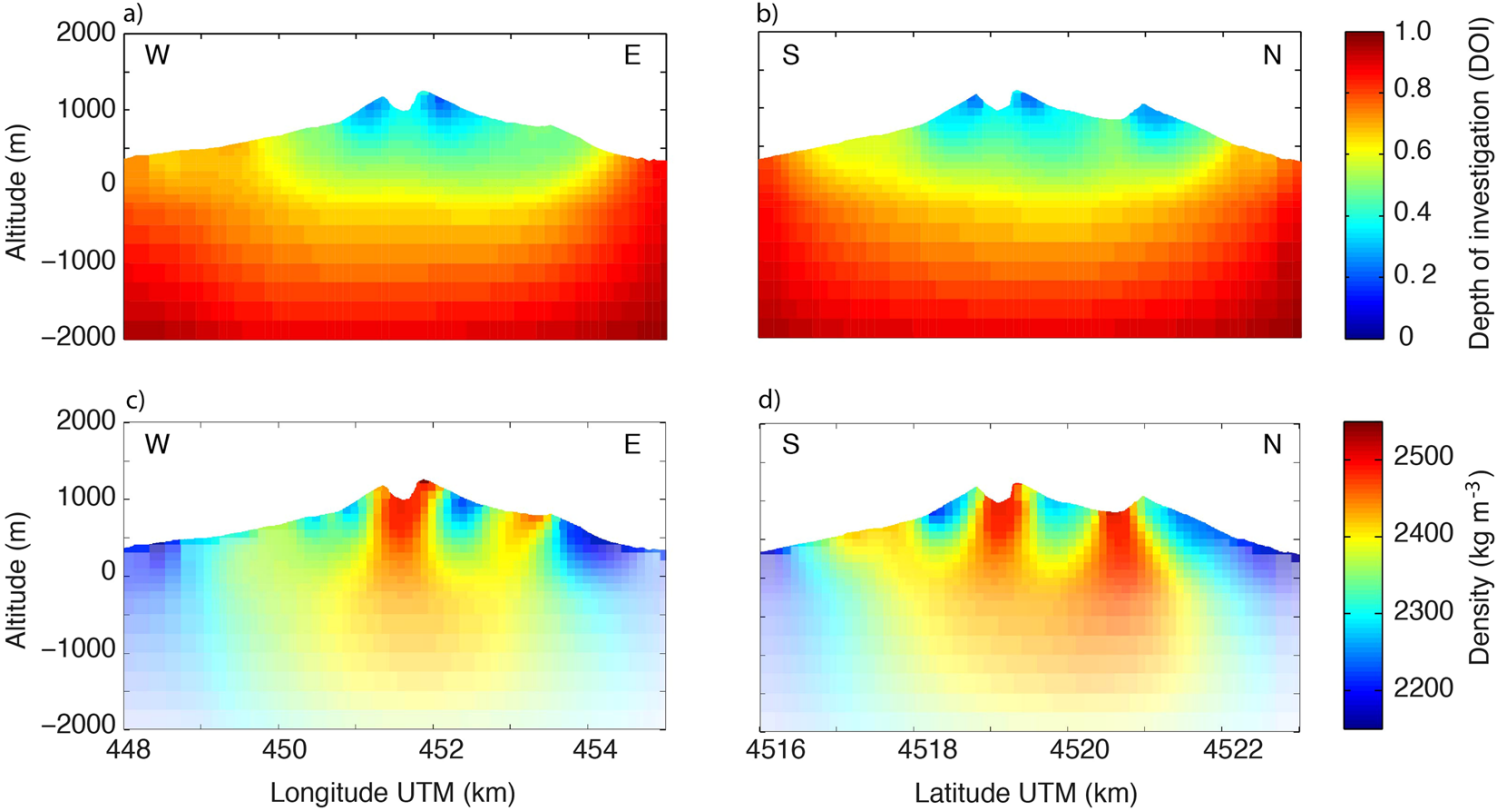
Depth of investigation (DOI) and vertical sections of the 3-D density model obtained with model regularization in terms of an isotropic roughness and damping constraints around 2200 kg m^−3^: (**a**) DOI for the west-east section at a latitude of 4519050 m and (**b**) the south-north section at a longitude of 451580 m; (**c**,**d**) corresponding density models. In (**c**,**d**), the DOI values are used to provide a qualitative (using transparency) assessment of how the model resolution decreases with depth. These sections also highlight how the inversion grid cells adapt to conform to the topography of the land surface at a resolution of 5 m. The locations of the two transects are highlighted in Fig. 1. The vertical sections were created using Matlab (version R2013b; www.mathworks.com).

To better appreciate the resolution of the gravimetric inversion results, we calculate the depth of investigation (DOI)^33^ based on inversion results with a 2000 kg m^−3^ and 2400 kg m^−3^ reference model, respectively. Two vertical slices (see Fig. 1) that cross in the Vesuvius crater demonstrate that the best-resolved region corresponds to the central part of the SVVC (Fig. 3a,b). In this region, the DOI is below 0.6, while the resolution is poor at deeper depths and increasing horizontal distances. Consequently, we mainly limit our discussion and interpretation to model features from the sea level and upwards.

**Figure 4:**
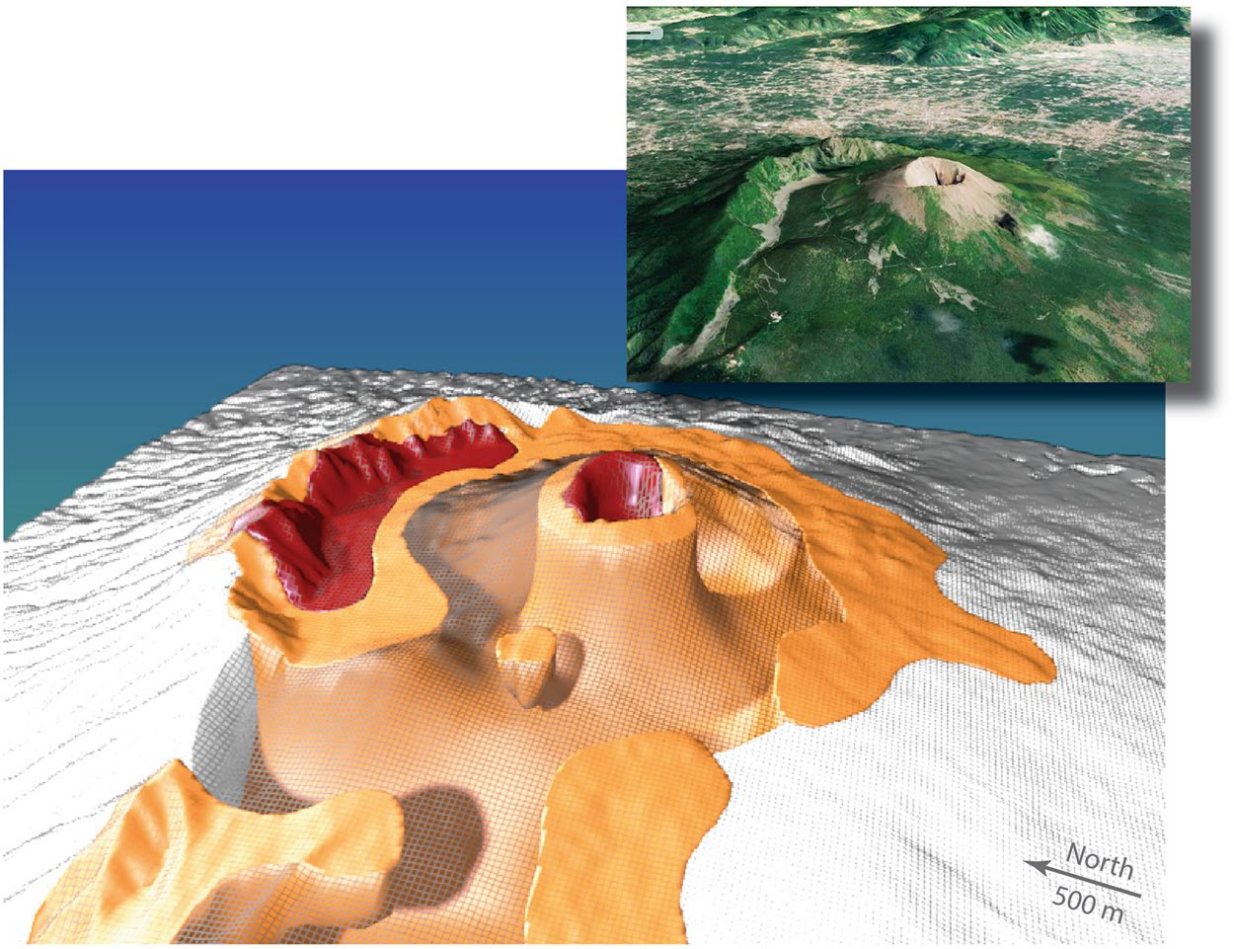
3D representation using Blender (version 2.78c; www.blender.org) of the comparatively low densities found between the Vesuvius cone and Mt. Somma. The rendered orange surface indicates a body with a density above 2370 kg m^−3^ and the denser red body a density above 2440 kg m^−3^. The present surface topography is represented by a black mesh and a satellite image from the same point of view is provided in the inset (Imagery ©2017, Map data ©2017 Google).

Two vertical sections (Fig. 3c,d) highlight some of the main features of the density model obtained from the gravity inversion. The region below the Vesuvius crater is markedly denser (>2450 kg m^−3^) than the surrounding flanks of the volcano (<2350 kg m^−3^). This central dense region appears continuous from the surface down to at least 2 km depth from the land surface, after which the model resolution is too poor to allow for definite statements about its continuation. Another prevalent feature is the dense body (>2400 kg m^−3^) that is found 1.5 km to the east (Fig. 3c) and to the north (Fig. 3d) of the Vesuvius crater. This dense body appears to be connected with the central dense body at depth, even if this cannot be confirmed given the poor model resolution at depth (Fig. 3c,d). The geometry of this central dense body is in strong agreement with seismic results (e.g., published figures: Fig. 7^24^, Fig. 10^23^ and Fig. 3^25^), as well as with aeromagnetic investigations (e.g., published figure: Fig. 10^26^). Its presence is also suggested by certain 3D gravity inversion results (published figures: Figs 6^21^ and 7^21^). A 3-D rendering of the dense regions (Fig. 4) highlights the geometry of the two dense bodies and the comparatively low densities below the flanks of the Gran Cono (Fig. 1).

## Discussion

**Figure 5:**
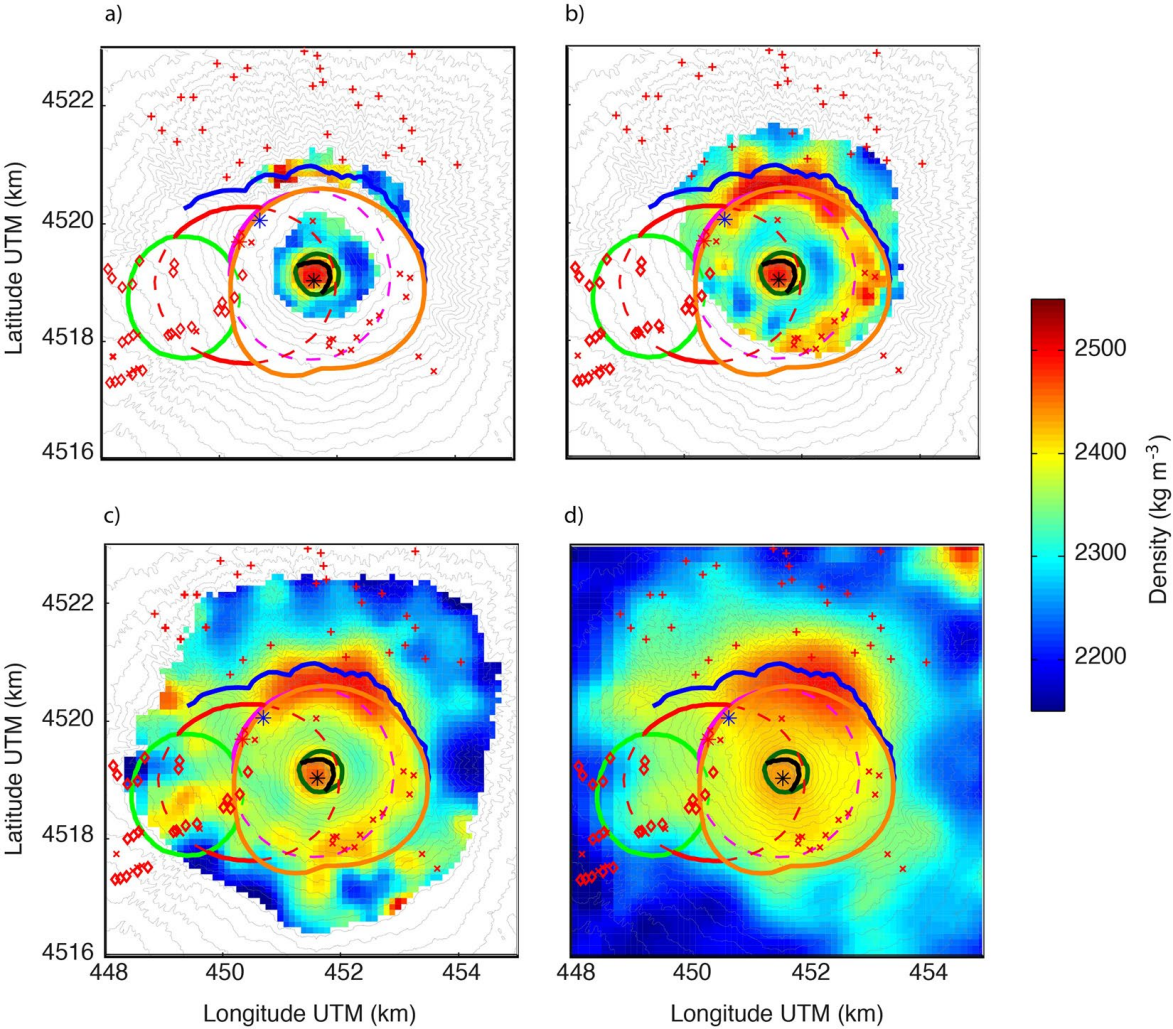
Horizontal slices of the 3-D density model at different altitudes with respect to the sea level: (**a**) 950 m, (**b**) 750 m, (**c**) 450 m and (**d**) −50 m. The overlain geological features are detailed in Fig. 1. The maps were created using Matlab (version R2013b; www.mathworks.com).

The geological interpretation is based on representative horizontal slices at decreasing altitudes. The slice at 950 m (Fig. 5a) displays a central roughly circular high-density feature corresponding to the inner part of Gran Cono (Fig. 1) and is delimited by the 1906 crater. We attribute the high-density values to the feeder conduit and to dense (low porosity) lava layers emitted at the beginning of the 20th century (1906–1915^34^; Fig. 119 in ref. 35) that overlap in correspondence to the buried rim. The porosity increases (density decreases) outside of the buried crater as lava flows are more scoriaceous and light ash, lapilli and scoriae deposits become more common. Moving to the north, a high-density feature is found that we attribute to the presence of abundant radial dikes and dense lava flows outcropping on the south wall in this sector of Mt. Somma^36, 37^.

At an altitude of 750 m (Fig. 5b), the high-density body is still positioned below the 1906–1944 tangent craters. It is surrounded by a roughly circular low-density body that hosts the shallow hydrothermal system of Mt. Vesuvius^38–40^. It consists of more porous material filling the depression created by the 1631 caldera-forming eruption, and in older times by the 79 AD Pompei eruption. Outside of this low-density region is a horseshoe-shaped high-density body that is partially open in the southwestern sector (this is also clearly seen in the 450 m slice in Fig. 5c). This denser body is related to the presence of dikes and old lavas belonging to the Mt. Somma edifice, whose upper part, reaching 1900 m a.s.l.^15^, was destroyed by two plinian caldera-forming eruptions (the Mercato eruption in the northwest to northeastern sector and the Pompei eruption in the northeast to southeastern sector)^14, 15^. South of the Mt. Somma scar, the persistent high density (showing up as a plateau in Fig. 4) is likely a manifestation of the accumulation of lava flows in the up to 400 m deep and originally narrow Atrio del Cavallo and Valle dell’Inferno valleys^41^ (Fig. 1). Outside of the horseshoe-shaped high-density body, the inferred densities decrease due to the increasing thickness of more porous products downslope^16^. Many of the parasitic vents located on the southeastern limit of the Mercato caldera are characterized by high-density values. In contrast to the general model of shallow and low-angle dike propagation at Vesuvius^42, 43^, our results rather suggest for most of those parasitic vents, as well as for the 1937 exogenous tholoid (Fig. 1), a deep and sub-vertical migration of magma from the source to the surface (c.f., Fig. 3c,d).

In the slice at 450 m (Fig. 5c), we still find the central conduit and the horseshoe-shaped dense body that marks the buried structures of the Mercato and Pompei calderas. On the northwestern rims of the Pompei and Mercato calderas, there is no clear high-density evidence of the conduits feeding the Pomici Verdoline vent, the 472 AD subplinian eruption^14^ or the 1895–1899 eruption of the Colle Umberto exogenous tholoid (Fig. 1). The lack of denser structures delimiting the Avellino caldera is explained by the fact that the remnants of that eruption are low-density tuffs originating from a giant tuff cone^15^. Within the Avellino caldera center, a denser body is seen that might be related to the shallow dike intrusion feeding the 1794 and 1861 eruptive parasitic vents located downslope and aligned along east-west striking fissures^16, 44, 45^. The high-density body located north of the Avellino caldera is explained by the accumulation, in a topographic depression, of thick layers of lavas erupted during 4 eruptions between 1855 and 1944^46^ and possibly in previous eruptive events. In the northern and eastern sectors of Mt. Somma, several radial higher-density bodies could indicate the presence of sub-horizontal radial dikes feeding presently buried parasitic vents developed more than 16.1 ka ago^47^.

The shape of the high and moderately high density region (green, yellow and red) in the −50 m slice (Fig. 5d) highlights the complex tectonic setting of the SVVC. The most pronounced feature is associated with an elongation in the northwest-southeast direction that corresponds to a major regional fault system (see. Fig. 2 in ref. 10). A similar elongation in the northeast-southwest direction coincides with another major regional fault system (see. Fig. 2 in ref. 10). These results underline how the development of the SVVC has been influenced by the tectonic Plio-Quaternary Campanian Plain depression that is buried 2 km below a polygenic filling^39^. Part of this filling surrounding the high-density body and laying on top of the Mesozoic carbonate basement^15, 48^ is represented by the 37 ka old deposit of Campi Flegrei Campanian Ignimbrite^49^. At this depth, the remnants of the ring structures produced by the four Plinian eruptions are either absent or unresolved.

We now discuss our results in terms of the hydrogeological setting of the SVVC, notably to provide constraints indicating if the SVVC is hydrogeologically connected to its surroundings or if it is closed by impervious caldera rims. This has important consequences in terms of groundwater geochemistry and flow. A hydrogeochemical study^50^ suggests that groundwater circulation within the SVVC is largely dependent on the volcano-tectonic structures^10, 48^ and the asymmetric topography of Mt. Somma^16, 51^. In the south-southwestern sector of Mt. Vesuvius, their sampled groundwater suggests strong interaction between volcanic aquifers and the main degassing system of the volcano, while no such intense interaction appears in groundwater samples from the Mt. Somma sector. This suggests that Mt. Somma acts as a barrier to groundwater flow in the northwestern sector. Mt. Vesuvius groundwater receives only a negligible contribution from Tyrrhenian seawater and thermal water, indicating an essentially meteoric origin and a predominant south-southwest flow direction. The proposed model of groundwater circulation (their Fig. 13^50^) is fully consistent with our horseshoe-shaped anomaly (Figs 4 and 5c,d) that suggests more porous and permeable subsurface deposits in the southwestern sector of the SVVC.

We now attempt to explain the main underlying reasons for the strong differences between our 3-D density model and previously published 3-D density models. A previous 3-D study^20^ at a similar scale and resolution as in our study led to a completely different density model. In that model, the region below the Vesuvius crater is comparatively light and the flanks of Gran Cono are comparatively dense. Given the expected positive relation between density and seismic velocity, these results were used to question seismic evidence^23–25^ of a high-velocity central quasi-spherical body below the crater surrounded by rocks of lower seismic velocities. We suggest that this discrepancy is rather explained by the use of a far too coarse DEM in this gravimetric study^20^. For example, by comparing our vertical profiles (Fig. 3c,d) with their corresponding figures (their Figs 12^20^ and 13^20^), it appears as if they do not account for the topography of the 300 m deep crater (i.e., the crater is absent in their profiles). This omission will inevitably force the smoothness-constrained inversion to compensate by introducing a large region of artificially low densities. It is also likely to introduce a surrounding halo of comparatively higher densities (in accordance with their results)^20^. Another illustration of the coarseness of their DEM is that they do not account for the strong topography of the Mt. Somma flank (compare the topography of their Fig. 12^20^ with the one in our Fig. 3d). Based on these arguments (and given that the results are inconsistent with extensive seismic investigations), we argue that this model^20^ is unreliable in regions affected by strong topography (i.e., throughout the SVVC).

Another study^21^ used a similar-sized model domain as in our study based on 400 of the historic data presented in Fig. 2a. The resulting density model is partly consistent with our results (e.g., a central high-density body and a structure that appears to be related with our horseshoe shaped dense body are found; see the slice at −1000 m in their Fig. 6^21^), but the results suffer from a low model resolution due to the very coarse station distribution in the central part of the SVVC (see Fig. 2a). If one would use their inversion approach with a finer discretization (possible today due to increased computing capabilities) and our new data, this would most likely make their results more comparable with our results. Nevertheless, their inversion cells do not conform to the surface topography, which leads to very limited information about the density distribution above the sea level (their presented depth slices are given from the sea level and downwards, while the peak of Mt. Vesuvius is at 1281 m a.s.l.). We stress that the focus of ref. 21 was placed on deeper structures and that it is largely complementary to our present study.

A more recent study^22^ resolves two dense bodies with locations that coincide with the northern and southern parts of the horse-shaped dense body. Unfortunately, this model appears to be adversely affected by the coarse model discretization used (815 m × 800 m in the horizontal dimensions compared to 100 m × 100 m with local adaptation to account for surface topography at a resolution of 5 m in our model). Such a coarse discretization will inevitably lead to a poor model resolution and the risk of introducing inversion artifacts. This may also explain the corresponding data misfit that is 7 times larger than for our inversion model.

An integrated modeling and inversion study that considered both seismic and gravimetric data^18^ constituted the first 3-D density model of the SVVC. The emphasis of that study was large-scale structures (e.g., they use interpolated gravity data every 2.5 km) and their 3-D model is based on the interpolation of 2-D results. Furthermore, their algorithm relies on the assumption of a strong and known relationship between seismic P-wave velocity and density. Despite these caveats, their model (their Fig. 8^18^) seems to indicate a denser body below the crater and lighter material on the sides. These results were improved by considering full 3-D modeling and inversion^19^ (albeit with a very coarse discretization of 2 km × 2 km × 0.5 km). The results confirm the presence of a thick and dense intrusion beneath the Vesuvius crater that they attribute to solidified dikes. An underlying assumed relationship between seismic velocity and density implies that this structure is not resolved by the gravity data alone.

## Methods

### Modeling and inversion framework

Our gravimetric data consist of relative variations of the vertical acceleration of gravity with respect to a base station (see Fig. 2b). These relative gravimetric data are mainly sensitive to the density distribution below the survey area. After appropriate data processing, it is possible to use them to obtain (by inversion) a 3-D density model. Traditionally, gravimetric data are interpreted in terms of density variations around a pre-defined background model. This is a sound approach for flat topography given that the acquired gravimetric data will then not carry any information about the mean density of the subsurface. Luckily, the pronounced topography of most volcanoes can be used to infer realistic density values (not only density variations). This was recently demonstrated at Stromboli volcano, Italy, in which a targeted processing flow and inversion provided, without any prior assumptions on density values, a density distribution that was in good agreement with density measurements on representative rock samples^31^. In this work, we largely follow this methodology^31^.

We discretize the SVVC and the surrounding region in terms of parallelepipeds and compute their forward responses using an analytic solution^52^. The parallelepipeds making up the central and interior part of the modeling domain are discretized with side-lengths of 100 m. Model cells that intersect the topography or the bathymetry of the Mediterranean Sea are refined to account for the topography at a spatial resolution of 5 m. The horizontal extent of the modeling domain over which topography is accounted for is 30 km × 30 km. The model cell dimensions grow towards the sides and with depth. A total of 564,988 model cells are used in the inversion.

There is an infinite number of density models that can explain a given gravimetric data set^53^. To obtain a unique solution, it is necessary to add penalties on how model parameters vary in magnitude and space (this is termed model regularization). Here, we rely on traditional isotropic roughness constraints in each spatial direction to ensure that the inferred density varies smoothly. Repeated inversions are performed (by trial-and-error) to find the regularization weight, *λ*, that provides a model with a forward response that explains the observed data to a user-defined error level. This procedure enables us to avoid obtaining a model that fits the data poorly (yielding a too smooth model) or a model that overfits the data (yielding a too rough model with inversion artifacts). Most inversion algorithms are based on a least-squares formalism^54^. After a first least-squares inversion step, we employ an additional iteration using iteratively reweighted least-squares^55^. By mimicking *l*_1_-norms (i.e., assuming underlying symmetric exponential distributions of the data misfit and model roughness), we decrease the sensitivity to data outliers and image sharper density contrasts. Compared with previous work at Stromboli volcano^31^, we also employ sensitivity scaling^56^ to enhance the imaging of structure at depth. This is achieved by scaling the regularization weight of every model cell to its accumulated sensitivity to all gravity data (i.e., the sum of absolute values). This ensures that model cells with a high data sensitivity will have strong roughness constraints. The sensitivity scaling leads to slightly lower density variations close to the surface (where the sensitivity is very high) and enhanced imaging of structures at larger depths. Nevertheless, the results are overall similar to those obtained without sensitivity scaling.

Another methodological addition is that we also incorporate a model regularization term that weakly penalizes deviations (so-called damping constraints) from a homogeneous pre-defined density model. By performing several inversions with different reference densities, we can assess to what extent the data (and not the model regularization) determines the inferred density values. This is achieved by using the concept of depth of investigation (DOI)^33^ that is widely used in geoelectrical studies to quantify model resolution in non-linear inversions. This approach is used herein to highlight the regions of the inversion model that are the most reliable. The DOI is obtained by performing two separate inversions with damping constraints around two different homogeneous reference models. The DOI for a given model cell is given by the difference between the two inversion results divided by the difference between the two reference models. A DOI of 0 suggests that the inferred density value is very well resolved (the inversion result is insensitive to the reference model), while a value of 1 suggests that the inferred density value is completely unresolved (the reference model wholly determines the inferred value).

We consider relative gravity data from which the influence of instrumental drift, earth tide, latitude, the acceleration of the sea mass for a known bathymetric model and the free-air effects have been removed^53^. The latitude and free-air corrections (associated with the normal gravity at the observation points) are made with respect to the reference station. This is done because our forward code provides relative responses of the density model with respect to the local reference, and not with respect to the normal reference ellipsoid. These local free-air anomalies with the sea effect removed that are used for inversion are only affected by the mass distribution below the discretized topographic surface^31^. We invert these residual anomalies on the topographic surface and account for the density distribution within our model domain that is bounded on the top by the irregular topography. This is the reason that we do not (and should not) make any topographic corrections to our data prior to inversion. The local Bouguer anomalies presented in Fig. 2 are not used for inversion, only for visualization. The inversion algorithm finds the most appropriate density value of each model cell together with parameters that describe a linear trend in the gravity data. This linear trend estimate is used to partly remove the influence of deep structures that we cannot resolve with our station distribution. The bathymetry is described by a bathymetric model with a resolution of 20 m^57^, while the land surface is described by an upscaled (5 m) version of a high-resolution (1 m) DEM obtained from LIDAR data (Digital Terrain Model by INGV-Osservatorio Vesuviano). This high-resolution DEM does not cover the whole area of interest and the topography of some of the external areas of the model domain is represented using the 20 m resolution DEM^57^.

### Data acquisition and processing

The new gravimetric data were acquired during the time period of October 5–20, 2014. The survey was designed to obtain a rather uniform distribution within a radius of 10 km from the Vesuvius crater with a refinement in the summit area and other areas of particular interest (see Fig. 2b). The relative gravimeter (CG-5, Scintrex) used returned the average response after measuring 30 s at 5 Hz. At each station, this sequence was repeated five times and the median value was retrieved for further analysis. The drift of the gravimeter was estimated by performing measurements at our local base station before and after the survey of each day. In addition, a network of additional reference stations was used to tie the measurements with the data from the previous days. The average drift correction each day was on the order or 0.03 mGal. The measurement locations and heights were obtained using DGPS (Topcon GR-5) and the local continuous GPS monitoring network^58^. It was sometimes difficult to obtain accurate positions for stations in the lower-lying areas located within pine forests. To improve the positioning capacity, we used the DGPS to search for station locations in the vicinity of the intended measurement point that had the best satellite configuration. The agreement between the DEM altitude and the one obtained by the DGPS was nevertheless not always good. To avoid inversion artifacts, we had to discard 25 stations with poor positioning accuracy. These points are mainly found in the forested landscape and it is likely that both the DGPS and the DEM positioning are less accurate in such regions. This leaves us with 297 new data points that were used in the subsequent inversion after the processing described above (free-air anomalies with sea effect removed).

The processed data set was merged with historic data^21^ that had previously been referenced to an absolute gravity station in Naples^59^. By occupying repeated points of the gravimetric monitoring network^60^, it was possible to link our relative measurements to the absolute gravity and, thus, to the historic data. The measurement locations of these old data are not very precisely determined as the data were acquired before the advent of DGPS. We assume uncorrelated data errors and that the combined errors of our new data have a mean deviation of 0.1 mGal. We assigned a mean deviation of 1 mGal for the historic data even if the actual errors might be slightly lower. For example, previous works suggest that the offshore data have a standard deviation below 0.7 mGal^32^ and the historic land-based data have been fitted to a standard deviation of 0.36 mGal^21^. Our reasoning for choosing the relatively high error level in the historic data was to force the inversion to resolve large-scale regional features and trends by including the larger survey area of the historic data (Fig. 2a), while ensuring that the new data (Fig. 2b) carry most of the weight in resolving the structure below the SVVC. For the new data, we have complete control and knowledge of the acquisition, positioning and processing.
